# Alteration of Membrane Cholesterol Content Plays a Key Role in Regulation of Cystic Fibrosis Transmembrane Conductance Regulator Channel Activity

**DOI:** 10.3389/fphys.2021.652513

**Published:** 2021-06-07

**Authors:** Guiying Cui, Kirsten A. Cottrill, Kerry M. Strickland, Sarah A. Mashburn, Michael Koval, Nael A. McCarty

**Affiliations:** ^1^Division of Pulmonology, Allergy/Immunology, Cystic Fibrosis, and Sleep, Department of Pediatrics, Emory + Children’s Center for Cystic Fibrosis and Airways Disease Research, Emory University School of Medicine and Children’s Healthcare of Atlanta, Atlanta, GA, United States; ^2^Division of Pulmonary, Allergy, Critical Care and Sleep Medicine, Department of Medicine, Emory University, Atlanta, GA, United States

**Keywords:** cystic fibrosis, cholesterol, modulator, potentiator, pharmacology, ivacaftor, Kalydeco

## Abstract

Altered cholesterol homeostasis in cystic fibrosis patients has been reported, although controversy remains. As a major membrane lipid component, cholesterol modulates the function of multiple ion channels by complicated mechanisms. However, whether cholesterol directly modulates cystic fibrosis transmembrane conductance regulator (CFTR) channel function remains unknown. To answer this question, we determined the effects of changing plasma membrane cholesterol levels on CFTR channel function utilizing polarized fischer rat thyroid (FRT) cells and primary human bronchial epithelial (HBE) cells. Treatment with methyl-β-cyclodextrin (MβCD) significantly reduced total cholesterol content in FRT cells, which significantly decreased forskolin (FSK)-mediated activation of both wildtype (WT-) and P67L-CFTR. This effect was also seen in HBE cells expressing WT-CFTR. Cholesterol modification by cholesterol oxidase and cholesterol esterase also distinctly affected activation of CFTR by FSK. In addition, alteration of cholesterol increased the potency of VX-770, a clinically used potentiator of CFTR, when both WT- and P67L-CFTR channels were activated at low FSK concentrations; this likely reflects the apparent shift in the sensitivity of WT-CFTR to FSK after alteration of membrane cholesterol. These results demonstrate that changes in the plasma membrane cholesterol level significantly modulate CFTR channel function and consequently may affect sensitivity to clinical therapeutics in CF patients.

## Introduction

Cystic fibrosis (CF) is caused by mutations in the gene encoding cystic fibrosis transmembrane conductance regulator (CFTR), a member of the ATP-binding cassette (ABC) Transporter Superfamily, but the sole member that functions as a chloride channel. CFTR bears two nucleotide binding domains (NBDs), twelve transmembrane helices (TMs), and a unique regulatory (R) domain. The level of PKA-mediated phosphorylation of the R domain is the main modulator of CFTR channel activity under physiological conditions (Cui et al., 2019b). CFTR functions as a chloride channel at the apical membrane of epithelial cells of multiple organ systems, including the airway. Lung failure caused by bacterial infections, inflammation, damage to airway tissue, and impairment of lung function is the major cause of mortality for CF patients. A number of small molecule therapeutics have been approved for use in CF patients, which partly improve patient quality of life and expand life expectancy (Wainwright, 2014; Wainwright et al., 2015; Joshi et al., 2019). The major CFTR-targeted therapeutics are categorized as correctors (affecting trafficking) and potentiators (affecting function). We and other researchers have found that the potentiator VX-770 (Ivacaftor, Kalydeco^TM^) potentiates CFTR channel activity in a manner dependent on CFTR’s phosphorylation level and also to varying degrees across specific CFTR mutants (Cui et al., 2016, 2019a,b; Veit et al., 2020). VX-770 is thought to bind to amino acids in the TMs (Y304, F312, and F931) to stabilize the open state of CFTR (Liu et al., 2019; Yeh et al., 2019).

Cholesterol is an essential constituent of cell membranes, comprising about 10–45% of the lipid bilayer in mammalian cells (Barbera and Levitan, 2019; Barbera et al., 2019). In addition, ∼80% of total cell cholesterol is in the plasma membrane (Endapally et al., 2019). Cholesterol not only contributes to controlling the membrane fluidity and lipid compartmentalization, but also modulates the function of a variety of membrane proteins, including the function of ion channels. Cholesterol mediates modulation via multiple mechanisms, including by direct binding to the targeted protein, or through affiliated subunits; cholesterol also modulates physical properties of the membrane, including fluidity (Barbera and Levitan, 2019; Barbera et al., 2019). For example, cholesterol exhibits an inhibitory effect on several K^+^ channels, including both the Kir_2.1_ inwardly rectifying K^+^ channel (Romanenko et al., 2004) and the Kv_1.5_ voltage-gated K^+^ channel (Abi-Char et al., 2007). Previous reports also suggest that cholesterol is involved in the regulation of the nicotinic acetylcholine receptor and the NMDA glutamate receptor (Barrantes, 2007; Korinek et al., 2015). Recently, Amsalem and coworkers reported that inflammation lowers cholesterol content in skin tissue and sensory neurons, and consequently enhances voltage-dependent activation of NaV_1.9_ channels leading to enhanced neuronal excitability (Amsalem et al., 2018).

The evidence that mutations in CFTR affect whole cell cholesterol metabolism has accumulated over years. For instance, CF patients appear to have generally low plasma cholesterol levels (Figueroa et al., 2002; Barnaby et al., 2019). Conversely, the loss of CFTR function increased the cholesterol content in the plasma membrane of cells expressing mutant CFTR. Interestingly, cholesterol was also found to modulate CFTR trafficking and mobility. For example, reduction of plasma membrane cholesterol content by methyl-β-cyclodextrin (MβCD) partially reduced internalization of F508del-CFTR in primary human airway epithelial cell cultures (Cholon et al., 2010). CFTR protein distribution in the plasma membrane was recently found to be grouped in two main populations. One population represented CFTR protein that was highly confined as a cluster in the lipid raft and its slow redistribution was highly dependent upon the cholesterol level. Cholesterol depletion decreased CFTR protein aggregation while elevating cholesterol increased CFTR aggregation in the lipid raft (Abu-Arish et al., 2015, 2019). However, the nature of the interaction between CFTR protein in the plasma membrane and membrane cholesterol remains unclear thus far. Furthermore, it is not clear whether CFTR channels that are localized to cholesterol-rich domains exhibit altered function compared to channels in the bulk membrane.

Since cholesterol and CFTR exhibit this reciprocal interaction, the question of whether cholesterol imbalance in CF patients affects CFTR function and pharmacology is directly relevant to the clinical outcome in patients. We took advantage of multiple approaches of modulating cholesterol levels in the plasma membrane to explore the impact of cholesterol on CFTR channel behavior in two cellular models: stably transfected Fischer Rat Thyroid epithelial cells (FRT) and primary human bronchial epithelial cells (HBEs). We found that CFTR activation by forskolin (FSK) and potentiation by VX-770 were significantly affected by modulation of plasma membrane cholesterol content with MβCD, cholesterol oxidase (CO), and cholesterol esterase (CE). We also found that modulation of plasma membrane cholesterol content in *Xenopus* oocytes altered the sensitivity of CFTR to inhibition by the pore-blocker, GlyH-101.

## Materials and Methods

### Fischer Rat Thyroid Cell Line and Primary Cells

Fischer rat thyroid cells stably expressing WT- and P67L-CFTR were kindly provided by the Sorscher lab (Sabusap et al., 2016). Cells were maintained in Coon’s modified Ham’s F12 medium at a liquid/liquid interface, supplemented with 5% fetal bovine serum, and grown at 37°C in a humidified incubator with 95% O_2_/5% CO_2_. Plating was at 1 × 10^6^ cells per well on 6.5 mm diameter Transwell permeable supports according to experimental requirements (Corning Inc., Corning, NY; catalog No. 3470). Primary human bronchial epithelial cells (HBEs) were obtained already seeded on Transwell permeable supports. HBE cells were maintained at an air / liquid interface and cultured as described (Stauffer et al., 2017). Methods for the growth of primary bronchial epithelial cells (HBEs) are based on work from the Randell laboratory. HBEs were isolated from human donor lung explants under an IRB-approved protocol through the CF@LANTA Experimental Models Core. The specific donor shown in this manuscript was female, age 45, with history of smoking for 16 years, but had not smoked for 10 years, with no active infection who was otherwise healthy. Costar #3470 plates (0.4 μm pore size, polyester, Corning) were used for plating. After 2 days on these Transwells, apical medium was removed, and cells were maintained at this air-liquid interface (ALI) to facilitate differentiation in medium based on the previously described ALI medium formulation (17). This medium included modifications to glucose (150 mg/dL; 8.3 mM), CaCl_2_ (1 mM), heparin (2 μg/ml), L-glutamine (2.5 mM), hydrocortisone 960 mg/ml, bovine pituitary extract (20 μg/ml), and Mg^2+^ (0.5 μM). Once cells were at ALI, the basolateral medium was changed every 48–72 h. Cultures were maintained in this manner at least 3 weeks to allow for differentiation.

### Ussing Chamber Analysis

Cystic fibrosis transmembrane conductance regulator-mediated transepithelial short-circuit Cl^–^ current (*I*_*sc*_) was measured under voltage clamp conditions (clamping voltage to 0 mV) as previously reported (Stauffer et al., 2017). The Ussing chamber system includes a VCC-MC6 amplifier and EM-RSYS-2 chambers, using P2302T sliders, with P2020-S electrodes (Physiologic Instruments, San Diego, CA, United States). Electrodes were prepared by adding about 1 cm of 3M KCl/3% agar to the electrode tips according to the manufacturer’s protocol. The voltage offset and fluid resistance compensation were set following manufacturer’s instructions with a blank filter in the chamber. Filters were inserted into the chamber and the following solutions added (in mM): basolateral solution (referred to as normal chloride solution) composed of 140 NaCl, 5 KCl, 0.36 K_2_HPO_4_⋅3H_2_O, 0.44 KH_2_PO_4_, 0.5 MgCl_2_, 1.3 CaCl_2_⋅2H_2_O, 4.2 NaHCO_3_, 10 glucose, 10 HEPES, (pH 7.4); apical solution composed of 133 Na Gluconate, 5 K Gluconate, 2.5 NaCl, 0.36 K_2_HPO_4_⋅3H_2_O, 0.44 KH_2_PO_4_, 0.5 MgCl_2_, 5.7 CaCl_2_⋅2H_2_O, 4.2 NaHCO_3_, 10 mannitol, and 10 HEPES (pH 7.4). Both solutions were bubbled with a 95/5% mixture of O_2_/CO_2_ and heated to 37°C. CFTR in FRT cells was activated by different concentrations of the adenylyl cyclase agonist forskolin (FSK). In HBE cells, amiloride (100 μM) was applied at the beginning of each experiment to block residual Na^+^ current from ENaC. *I*_*sc*_ of HBE cells was recorded in symmetrical 140 mM NaCl normal chloride solution. CFTR_*inh*_172 (10 μM) was administered to the apical solution at the completion of each experiment to block CFTR-dependent *I*_*sc*_. Data were acquired using the Acquire and Analyze software and exported to Excel for analysis.

### Filipin III Staining

Fischer rat thyroid cells were cultured on cover slips coated with collagen I (20 μg/ml) for 2–4 days. Cells were washed with PBS solution and treated with 5 mM MβCD diluted in normal chloride solution for 1 h in the cell culture incubator. Control cells were treated with 5 mM mannitol diluted in normal chloride solution for 1 h in the cell culture incubator. Cells were washed with PBS solution once, fixed in 4% paraformaldehyde for 20 min, then washed three times with PBS solution for 5 min each time. Cells were then stained for 1 h with 10 μl/ml filipin III (Sigma, SAE0087, 10 mg/ml in DMSO) diluted in PBS in the dark. Cells were washed with PBS two times for 5 min each time, then mounted with Vecta Shield without DAPI. Images were taken (at 20 X) using a fluorescence microscope with a UV filter (Integrated Cellular Imaging Core, Emory University). The filipin intensity was calculated with ImageJ.

### Mass Spectrometry Analysis of Cholesterol

Fischer rat thyroid cells were cultured in 60 mm cell culture dishes for 3 days. Cells were washed with PBS and treated with 5 mM MβCD diluted in normal chloride solution for 1 or 2 h in the cell culture incubator. Control cells were treated with 5 mM mannitol diluted in normal chloride solution for the same duration as the MβCD group. The following Mass Spectrometry analysis was done by Georgia Institute of Technology’s Systems Mass Spectrometry Core Facility. Cells were washed with PBS and detached with 0.25% trypsin/EDTA, then collected and spun down. The supernatant was removed. Cells were washed once with PBS. Cell pellets were stored in the −80°C freezer for subsequent Mass Spectrometry analysis. Samples were removed from the −80°C freezer and thawed on ice. A 1 ml aliquot of isopropyl alcohol (IPA) containing 5 μM deuterated cholesterol [Chol(d7)] was added to each sample, except the blank, which received exclusively IPA. Samples were submerged in liquid nitrogen until frozen and then thawed and sonicated in an ice bath for 3 min. This freeze-thaw cycle was repeated three times. Samples were centrifuged at 10,000×*g* for 8 min. A pool was made from 100 μL aliquots from each sample. The pool and samples were transferred to liquid chromatography (LC) vials and stored at 4°C until analysis. Reverse Phase Column: Accucore C30, 2.6 μm, 150 × 2.1 mm; Mobile Phase A: 40% H_2_O/60% ACN (acetonitrile), 10 mM ammonium formate, 0.1% FA (Formic Acid); Mobile Phase B: 10% ACN / 90% IPA, 10 mM ammonium formate, and 0.1% FA; Column Temperature–50°C, 2 μl sample injection.

### Expression in *Xenopu*s Oocytes

Human CFTR cRNA used in this study was prepared from a construct encoding the WT-CFTR gene in the pGEMHE vector. *Xenopus laevis* oocytes were injected with ∼10 ng of CFTR cRNA, and were incubated at 17°C in modified Liebovitz’s L-15 media (pH 7.5) with penicillin, and streptomycin. Experiments were performed 24 - 96 h following injection of cRNAs. Protocols for *Xenopus laevis* frog handling and oocyte collection were based on and adhere to NIH guidelines, and have been approved by the Institutional Animal Care and Use Committee of Emory University (Cui et al., 2019b).

### Source of Reagents

Unless otherwise noted, all reagents were obtained from Sigma Chemical Co. (St. Louis, MO, United States). Cholesterol Esterase was purchased from Millipore. CFTR_*inh*_172 was purchased from Calbiochem (Burlington, MA, United States) and prepared as 50 mM stock in DMSO. VX-770 was obtained from Selleckchem (Houston, TX, United States) and was initially prepared as a stock solution at 10 mM in DMSO. All chemicals were diluted to a final concentration in experimental solution immediately prior to use.

### Data Analysis

Unless otherwise noted, values given are mean ± SEM. Statistical analyses were performed using the *t*-test for unpaired measurements in Sigmaplot 12.3 (San Jose, CA, United States). *P* < 0.05 was considered significantly different. ^∗^*P* < 0.05; ^∗∗^*P* < 0.01; ^∗∗∗^*P* < 0.001.

## Results

### Methyl-β-Cyclodextrin Efficaciously Extracts Cholesterol From the Plasma Membrane of FRT Cells

Because cholesterol is very important in maintaining cell integrity and activity, cells may be very sensitive to the effects of manipulated cholesterol levels in the plasma membrane. In other words, the removal of too much cholesterol could be lethal for the cell, while the removal of too little cholesterol could have undetectable effects in the conducted experiments. Cyclodextrins such as MβCD were previously found to extract cholesterol from membranes, reducing both accessible and total cholesterol in the plasma membrane (Kinnebrew et al., 2019). We chose to test the effects of cyclodextrin-mediated reduction of cholesterol content on WT-CFTR as well as the rare mutant P67L-CFTR for the following reason. P67L is in the Lasso motif of CFTR (specifically, in Elbow Helix #1) and is identified as both a type II (trafficking defect) and a type III (gating defect) mutation. The Lasso motif is thought to localize within the hydrophobic membrane bilayer, so may be more sensitive to membrane composition. We previously reported that P67L-CFTR exhibits different PKA sensitivity compared to WT-CFTR and suggested that the gating defect of P67L may be due to a break in the interaction between the Lasso motif and the R domain (Cui et al., 2019b).

**FIGURE 1:**
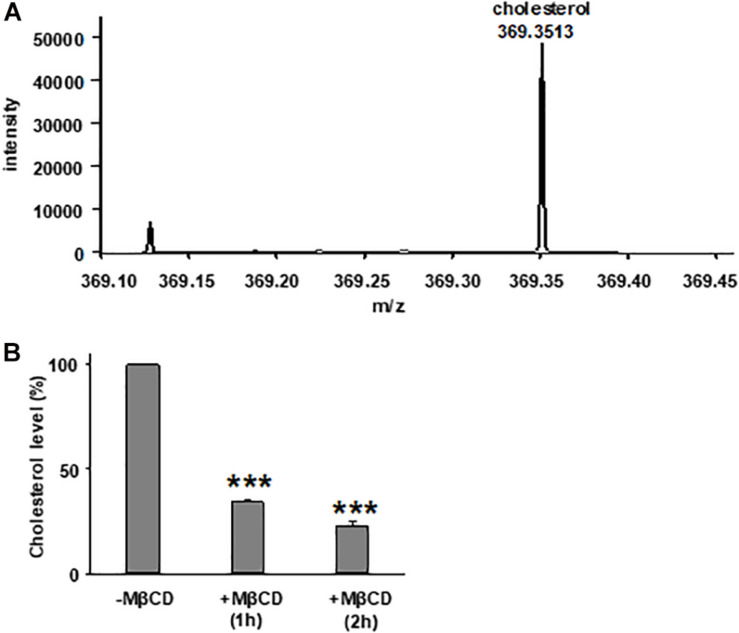
Treatment with methyl-β-cyclodextrin (MβCD) reduces total cholesterol level in Fischer rat thyroid (FRT) cells. **(A)** A representative extracted ion chromatogram for *m/z* 369.3513 (cholesterol) from mass spectrometry analysis. **(B)** Total cholesterol level of FRT cells in control conditions (-MβCD, 5 mM mannitol) and after 5 mM MβCD at 37°C (1 and 2 h). *n* = 7 for control group; *n* = 3 for 1 h group; and *n* = 4 for 2 h group. ***P < 0.001 compared to control group.

**FIGURE 2:**
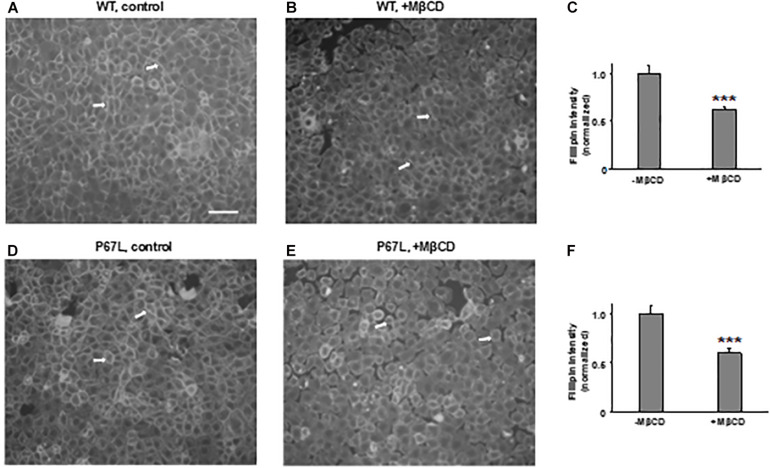
Methyl-β-cyclodextrin efficaciously extracts cholesterol from the plasma membrane of FRT cells. Representative images for cholesterol staining with filipin III in FRT cells expressing WT- **(A,B)** or P67L-CFTR **(D,E)**. Cells were cultured on collagen-coated cover slips for 24–48 h. Cells then were treated without (control, 5 mM mannitol, -MβCD) and with 5 mM MβCD at 37°C for 1 h. Scale bar: 100 μm. Arrows: the plasma membrane with filipin III staining. Normalized filipin intensity was analyzed with ImageJ for cells expressing WT-CFTR **(C)**, and P67L-CFTR **(F)**. n = 6 for each. ***P < 0.001 compared to control (-MβCD).

We first tested the reduction of total cholesterol in polarized FRT cells by MβCD utilizing Mass-Spectrometry (Figure 1) and confirmed the time-dependent decrease in the plasma membrane cholesterol level by immunostaining (Figure 2) (Zidovetzki and Levitan, 2007). As shown in Figure 1, total cholesterol changes were measured with Mass-Spectrometry of FRT cell expressing WT-CFTR, indicating reduction of total cholesterol after treatment with 5 mM MβCD for 1 or 2 h at 37°C. Total cholesterol was reduced over 50% in a time-dependent manner. We also stained FRT cells with antibody against fillipin III in order to characterize the change in plasma membrane cholesterol level caused by MβCD. As shown in Figure 2, 5 mM MβCD, but not 5 mM mannitol (control), significantly reduced plasma membrane cholesterol in the FRT cells expressing WT- or P67L-CFTR after a 1 h treatment.

### Methyl-β-Cyclodextrin Treatment Affects Activation of CFTR Channels by Forskolin

**FIGURE 3:**
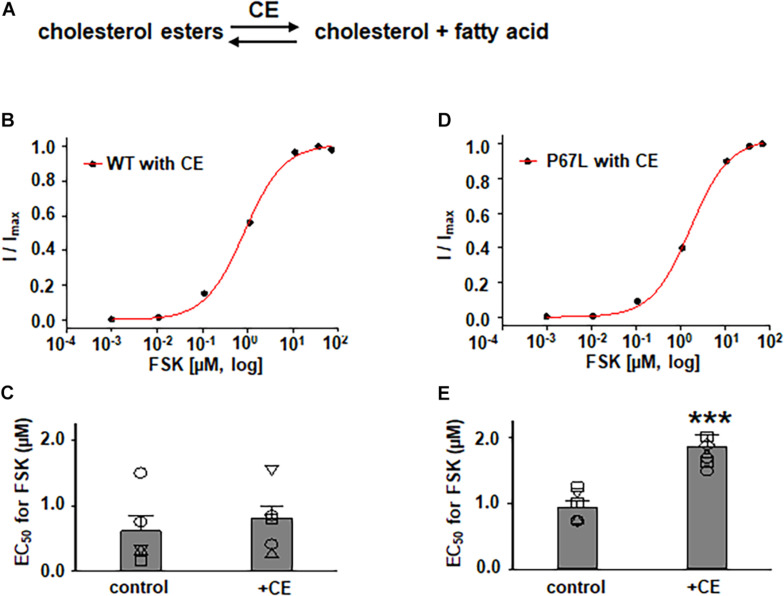
Effects of cholesterol esterase (CE) on WT- and P67L-CFTR expressed in FRT cells. **(A)** CE catalyzes the conversion of cholesterol esters to cholesterol and fatty acid. CE was added to both apical and basolateral sides of the Ussing chamber for 1 h at 37°C before performing experiments. WT- and P67L-CFTR currents were activated with various concentrations of FSK as shown in Figures 4, 5 after pretreatment with 10 U/ml cholesterol esterase at 37°C for 1 h. Dose-response curves for representative normalized short circuit currents [**(B)** WT-CFTR, treated with CE; **(D)** P67L-CFTR, treated with CE] were fit with the one site ligand binding equation in Sigmaplot 12.3. Summary data for half-maximal activation concentrations (EC_50_) for FSK are shown in panel **(C)** for WT-CFTR and **(E)** for P67L-CFTR. ***P < 0.001 compared with control condition. *n* = 5–7 for each group.

**FIGURE 4:**
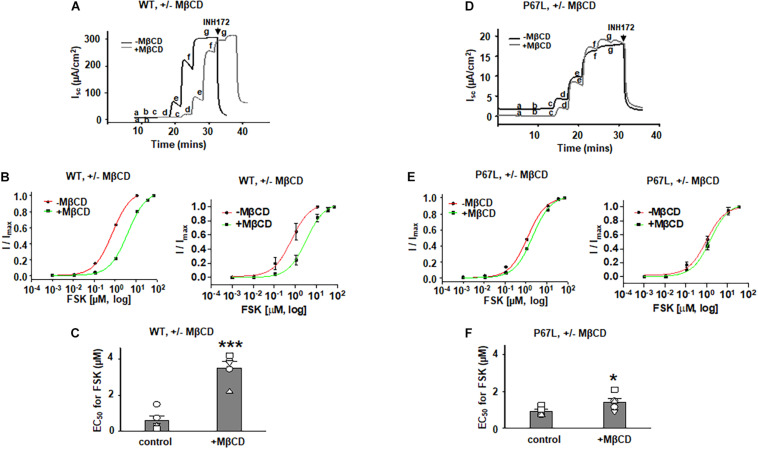
Methyl-β-cyclodextrin pretreatment of FRT cells affected activation of Cystic Fibrosis transmembrane conductance regulator (CFTR) by forskolin (FSK). 5 mM MβCD was added to both apical and basolateral sides of the Ussing chamber. Representative short-circuit current traces for wildtype (WT)- **(A)** and P67L-CFTR **(D)** activated with varied concentrations of FSK with or without pretreatment with 5 mM MβCD at 37°C for 1 h. Currents were inhibited by 10 μM CFTR_*inh*_172 (INH172) at the end of the experiment. FSK concentrations for experiments in treated and untreated cells in both **(A,D)** are labeled as follows: *a* = 1 nM; *b* = 11 nM; *c* = 111 nM; *d* = 1 μM; *e* = 11 μM; *f* = 36 μM; and *g* = 71 μM. Both FSK and INH172 were added to the apical side of the Ussing chamber. Representative normalized currents of WT- **(B)** and P67L-CFTR **(E)** were fit with the one site ligand binding equation in Sigmaplot 12.3. In both panel **(B,E)**, a representative single experiment is shown at *left*, while the summary data (mean +/- standard deviations) are shown at *right*. Summary data for half-maximal activation concentrations (EC_50_) for FSK with and without MβCD treatment are shown in panel **(C)** (WT-CFTR) and **(F)** (P67L-CFTR). ***P < 0.001 compared to control condition. *P < 0.05 compared to control condition. n = 5–7 for each group.

To determine the effects of plasma membrane cholesterol depletion on CFTR activity, we studied FSK-activated short-circuit currents in FRT cells using the Ussing chamber recording technique. As noted above, we treated FRT cells with 5 mM MβCD for 1 h at 37°C. Representative current traces of WT- and P67L-CFTR activated by FSK are shown in Figures 3, 4A,D. After MβCD treatment, the FSK concentration-dependent activation curve shifted to the right for both WT- and P67L-CFTR (Figures 4B,E). The calculated FSK concentrations for half-maximal activation (EC_50_) were significantly increased in both WT- and P67L-CFTR after MβCD treatment (Figures 4C,F). These data suggest that reduction of cholesterol from the plasma membrane of FRT cells significantly reduced the potency of FSK for activating both WT- and P67L-CFTR, as if the altered interaction with cholesterol shifted CFTR into a state less amenable to activation.

### Effects of Cholesterol Oxidase and Cholesterol Esterase on Activation of CFTR Channels Expressed in FRT Cells

**FIGURE 5:**
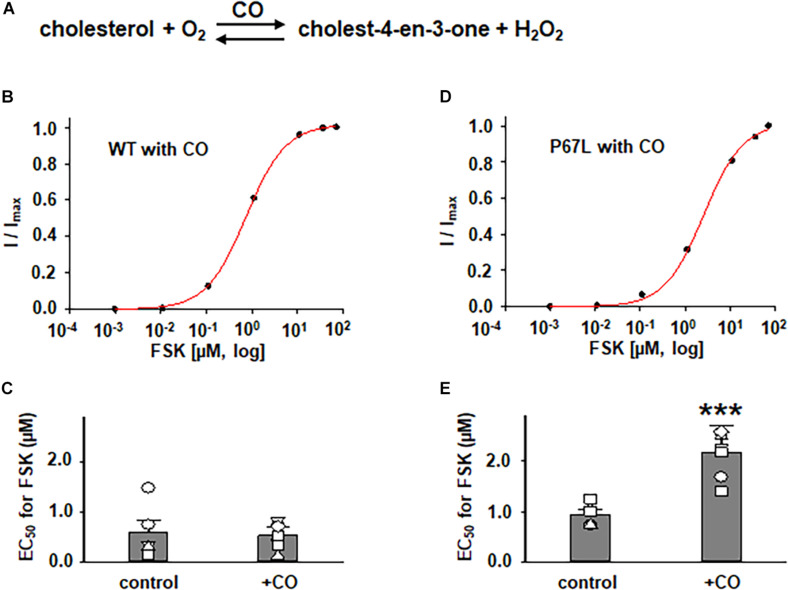
Effects of cholesterol oxidase (CO) on WT- and P67L-CFTR expressed in FRT cells. **(A)** CO catalyzes the conversion of cholesterol and oxygen to cholest-4-en-3-one and hydrogen peroxide. CO was added to both apical and basolateral sides of the Ussing chamber for 1 h at 37°C before performing experiments. Dose-response curves for representative normalized short circuit currents [**(B)** WT-CFTR, treated with CO; **(D)** P67L-CFTR, treated with CO] were fit with the one site ligand binding equation in Sigmaplot 12.3. Summary data for half-maximal activation concentrations (EC_50_) for FSK are shown in panel **(C)** for WT-CFTR and **(E)** for P67L-CFTR. ***P < 0.001 compared with control condition. n = 5–7 for each group.

In addition to exploring the effects of reducing plasma membrane cholesterol content using MβCD, we further asked whether enzymatic modification of cholesterol may also exhibit effects on CFTR channel activation by FSK. Prior studies show that both CO and CE significantly affect CFTR protein diffusion in the plasma membrane of HBE cells (Abu-Arish et al., 2015, 2019). CO catalyzes cholesterol and O_2_ to cholest-4-en-3-one and H_2_O_2_, disrupting lipid rafts (Kinnebrew et al., 2019) (Figure 5 and Supplementary Figure 1). We treated FRT cells with CO (5 U/ml) at 37°C for 1 h and tested the concentration-dependent activation of WT- and P67L-CFTR by FSK. CO treatment had no significant effect on WT-CFTR but caused the EC_50_ for FSK to significantly increase in P67L-CFTR. As opposed to CO, CE hydrolyzes cholesterol esters to unesterified cholesterol and free fatty acids (Figure 3) (Abu-Arish et al., 2015). We treated FRT cells with CE (10 U/ml) at 37°C for 1 h and tested the concentration-dependent activation of WT- and P67L-CFTR by FSK. The EC_50_ for FSK-mediated activation remained unchanged by CE in WT-CFTR, while CE significantly increased the EC_50_ for FSK in P67L-CFTR. These two groups of data suggest that not only MβCD but also CO and CE significantly modulate cholesterol levels in the plasma membrane, and this consequently affects CFTR channel activity. Moreover, while WT-CFTR was more sensitive to MβCD-induced reduction in cholesterol content than P67L-CFTR, the latter appears to be much more sensitive to cholesterol modification by CO and CE, perhaps due to the likelihood that this mutation in the Lasso motif impacts stability of CFTR protein at the membrane interface (Bozoky et al., 2013; Cui et al., 2019b).

The differential sensitivity of P67L-CFTR to alterations of cholesterol content by extraction vs. enzymatic means suggests that the chemical structure of cholesterol may be important to this modulatory effect. This may provide useful information in localizing the functionally relevant cholesterol binding site. In addition, the data suggest that cholesterol might directly interact with CFTR protein, and may as well as propose the possibility of multiple binding sites involved in the interaction between cholesterol and the CFTR channel. Moreover, the data also suggest that both an increase (by CE) and decrease (by MβCD or CO) of the plasma membrane cholesterol made P67L-CFTR channel activation by FSK more difficult, consequently maintaining the plasma membrane cholesterol at physiological levels was the best condition for maximizing CFTR activation. In other words, cholesterol level and CFTR function have a tight relationship in maintaining CFTR channel behavior.

### Changing Membrane Cholesterol Level Modulates Potentiation of CFTR by VX-770

**FIGURE 6:**
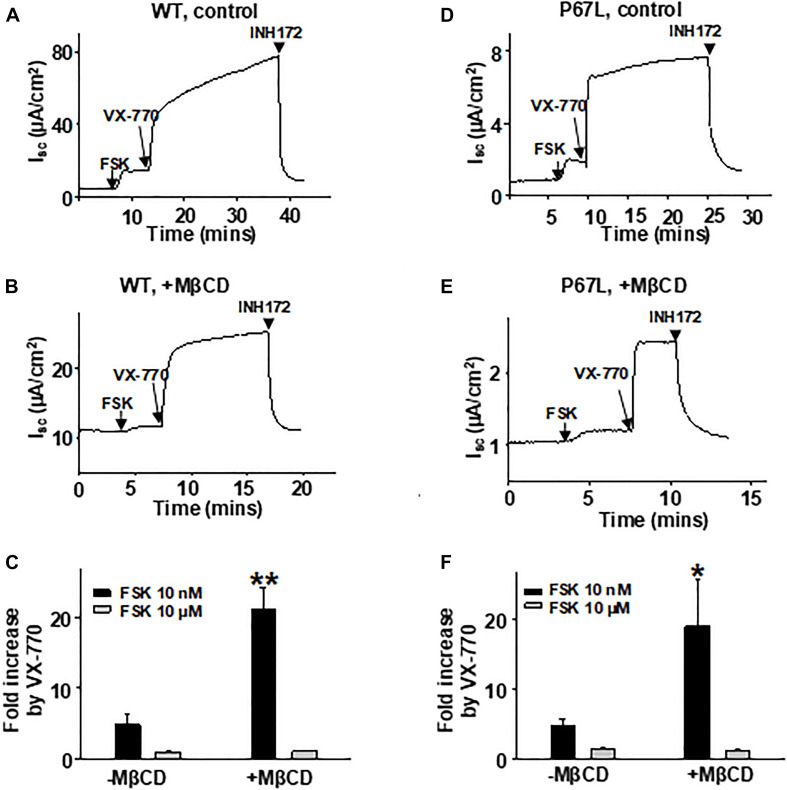
VX-770-mediated potentiation of CFTR was affected by plasma membrane cholesterol depletion with MβCD. 5 mM MβCD was added to both apical and basolateral sides of the Ussing chamber. 1 μM VX-770 potentiated WT- and P67L-CFTR differently in control conditions (-MβCD) compared to after pretreatment with MβCD (+MβCD). The FRT cells were pre-incubated with 5 mM MβCD at 37°C for 1 h prior to experimentation. Representative short circuit currents of WT-CFTR **(A,B)** and P67L-CFTR **(D,E)** activated with 10 nM FSK and further potentiated with 1 μM VX-770 recorded with the Ussing chamber technique. Channels were also tested when activated with FSK at high concentration (10 μM) followed by addition of 1 μM VX-770 in the continuing presence of FSK. CFTR currents then were inhibited with 10 μM INH172. Summary data for fractional increase of WT- **(C)** and P67L-CFTR **(F)** current in response to VX-770 under each set of conditions are shown. *n* = 5–7. **P < 0.01 compared to control; *P < 0.05 compared to control.

We have previously shown that the efficacy of VX-770-mediated potentiation of CFTR channel activity is dependent upon the phosphorylation level (Cui et al., 2019b), indicating that the efficacy of VX-770 is larger on channels that are in a state less amenable to opening. Here, we found that modulation of cholesterol levels in the plasma membrane affected the concentration-dependent activation of CFTR channels by FSK, suggesting that reduction in cholesterol content shifted CFTR into a state less amenable to activation. Because VX-770 generally exhibits greater efficacy in potentiating, or repairing, the activity of plasma membrane-resident CFTR channels that are defective in opening (i.e., Type II mutants) (Cui et al., 2019b), we asked whether modulating the plasma membrane cholesterol level with MβCD affected the potency of VX-770-mediated potentiation. We treated FRT cells expressing WT- or P67L-CFTR with 5 mM MβCD for 1 h at 37°C, then activated CFTR channels with low (10 nM) or high (10 μM) concentration of FSK, and finally added 1 μM VX-770 to potentiate CFTR activity (Figure 6). The effect of VX-770, presented here as fold-increase in FSK-activated current, was significantly increased after MβCD treatment in both WT- and P67L-CFTR when the channels were activated by FSK at low concentration (10 nM). However, MβCD treatment failed to affect potentiation by VX-770 when channels were activated with FSK at high concentration (10 μM); this may be related to the “ceiling effect” phenomenon that we previously described for CFTR, which suggests that supramaximal phosphorylation of CFTR *in vitro* makes it less sensitive to modulating factors such as both VX-770 and cholesterol (Cui et al., 2019b). In contrast, since the CFTR phosphorylation level in cells under physiologically relevant conditions *in vivo* is likely to be very low (Cui et al., 2019b), these data suggest that changes in the cholesterol level in the plasma membrane could potentially affect the response of CFTR channels to VX-770 in airway cells of CF patients, including those bearing rare mutation genotypes.

### Changing Membrane Cholesterol Level Affects CFTR in Primary Airway Cells and Oocytes

**FIGURE 7:**
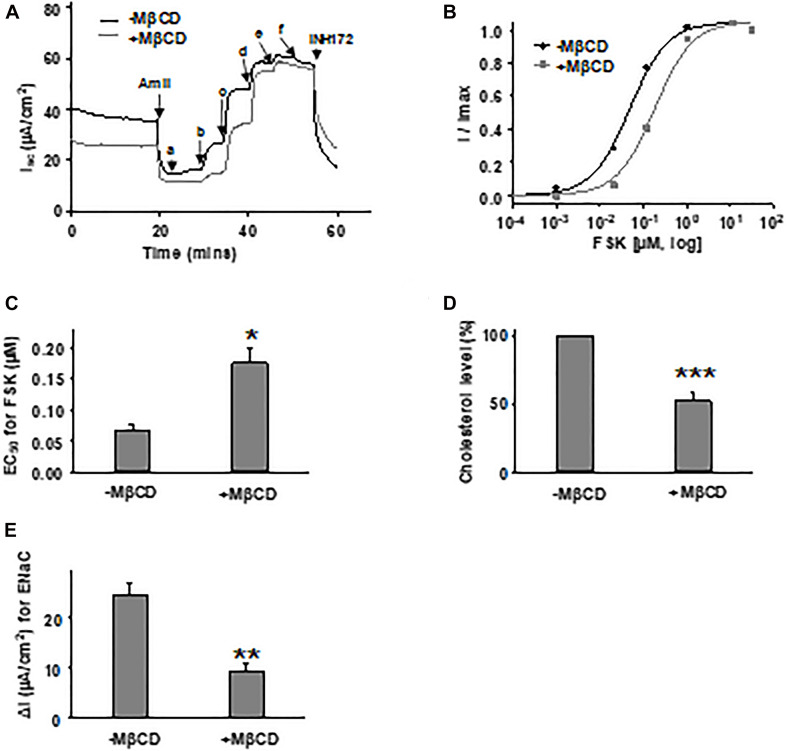
Forskolin-mediated activation of WT-CFTR in human bronchial epithelial (HBE) cells was influenced by cholesterol depletion with MβCD. 5 mM MβCD was added to both apical and basolateral sides of the Ussing chamber. Representative currents of WT-CFTR in the control condition (-MβCD) and after pretreatment with 5 mM MβCD (+MβCD) (37°C, 1 h), then activated by different concentrations of FSK **(A)**. FSK concentrations are labeled as follows: *a* = 1 nM; *b* = 11 nM; *c* = 111 nM; *d* = 1 μM; *e* = 11 μM; and *f* = 51 μM. Currents then were blocked by 10 μM INH172. Representative normalized currents **(B)** were fit with the one site ligand binding equation with Sigmaplot 12.3. Summary data for FSK EC_50_ are shown in panel **(C)**. *P < 0.05 compared with control condition. *n* = 5 for both groups. **(D)** HBE cells in the MβCD treated group exhibited significantly lower total cholesterol levels compared to control group, as measured with mass spectrometry. ***P < 0.001 compared to control group. *n* = 5 for (-MβCD) and *n* = 6 for (+MβCD). **(E)** Epithelial sodium channel (ENaC) current is significantly reduced in HBE cells with MβCD treatment (5 mM MβCD, 37°C, 1 h) compared to the untreated group (-MβCD), as recorded with the Ussing chamber technique. ^∗∗^*P* < 0.01 compared to control group. *n* = 5 for (-MβCD) and *n* = 6 for (+MβCD).

To rule out the possibility of cell model-dependent effects and to study CFTR channel behavior in more physiologically relevant conditions, we further tested FSK concentration-dependent activation of WT-CFTR expressed in primary bronchial epithelial cells (HBEs) after treatment with 5 mM MβCD for 1 h at 37°C (Figures 7A–C). The EC_50_ for FSK-mediated activation was significantly increased after MβCD treatment which is similar to the effects seen in FRT cells. Treatment of HBE cells with 5 mM MβCD for 1 h at 37°C reduced the total cholesterol by ∼50% as measured with mass spectrometry, again very similar to results seen in FRT cells (Figure 7D). Studying CFTR expressed heterologously in *Xenopus* oocytes, we also found that block of WT-CFTR by the extracellular open pore blocker GlyH-101 was enhanced by reduction of cholesterol content with MβCD (Supplementary Figure 2), suggesting that loss of cholesterol modifies pore architecture. These data suggest that decreasing the plasma membrane cholesterol level affects CFTR in a manner that is not dependent upon cell type. While this study has focused on the direct effects of changing cholesterol level on CFTR function, we also found that amiloride-sensitive current mediated by the epithelial sodium channel (ENaC) was significantly decreased with MβCD treatment in HBE cells (Figure 7E), consistent with previous reports (Krueger et al., 2009; Zhai et al., 2018). Along with CFTR, ENaC also is an important player in the development of CF disease; therefore, the cholesterol imbalance in CF patients could affect the behavior of multiple proteins.

## Discussion

The study presented here found that alteration of the plasma membrane cholesterol level by different methods led to the modification of CFTR channel behavior, including its activation by FSK, potentiation by VX-770, and block by GlyH-101. The results suggest that various forms of CFTR may differ in their cholesterol-mediated modulation, because P67L-CFTR clearly exhibited different reactions to cholesterol modulation compared to WT-CFTR in multiple ways. In addition, Chin and colleagues recently reported that unlike CFTR protein purified in detergent, CFTR purified with phospholipids and cholesterol exhibited alterations in ATPase activity, phosphorylation dependent channel activation, and potentiation by VX-770 (Chin et al., 2019). Taken together, the findings thus far suggest a specific role for cholesterol in modulating CFTR activity since addition of phospholipids alone failed to rescue CFTR protein activity (Liu et al., 2017, 2019; Chin et al., 2019).

The accumulated evidence suggests two potential interactions between cholesterol metabolism and CFTR mutations (Cottrill et al., 2020). One proposed mechanism is that CFTR mutation causes a defect in cholesterol metabolism. In support of this, it was reported previously that mutant CFTR decreases lipoprotein abundances and alters cholesterol metabolism (Vaughan et al., 1978; Worgall, 2009). Meanwhile, CF patients have low plasma HDL, LDL, and total cholesterol levels (Vaughan et al., 1978; Worgall, 2009). In addition, cholesterol accumulation was found in CF model cells and free cholesterol is increased in cells from CFTR knockout mice and epithelial cells from CF patients. The data together suggest an inherent defect in intracellular cholesterol transport in CF cells which is not limited to those expressing F508del-CFTR (White et al., 2004, 2007). On the other hand, changes in cholesterol levels in the plasma membrane not only affected CFTR protein localization but also modulated CFTR channel activity (Abu-Arish et al., 2015, 2019). Cholesterol also was found to bind to the PDZ-domain of NHERF1/EBP50 to significantly increase the plasma membrane localization and dwell time of WT-CFTR protein (Sheng et al., 2012). Our data presented here suggest that cholesterol could bind to CFTR and directly modulate its channel activity in a mutation-specific manner. Cholesterol may bind to CFTR in varied locations and consequently removal of cholesterol-CFTR interactions by mutation at different locations could have different effects on CFTR channel behavior. Furthermore, the data showed that reduction of plasma membrane cholesterol in FRT cells significantly affected potentiation efficiency of VX-770, which may be clinically relevant.

In addition to our results on efficacy of VX-770, changing the cholesterol level could also affect the function of the CFTR corrector, VX-809. Barnaby and coworkers recently reported that outer membrane vesicles (OMVs) isolated from *Pseudomonas aeruginosa* inhibited Cl^–^ secretion by F508del-CFTR after rescue by VX-809, while apical treatment of cells by hydroxypropyl-β-cyclodextrin (HPβCD) and MβCD prohibited the effects of OMS on inhibition of Cl^–^ secretion (Francis et al., 1999; Nusrat et al., 2000; Barnaby et al., 2019). Lu and coworkers found that chronic VX-809 treatment of F508del-CFBE cells reduced plasma membrane cholesterol content but this effect was lost in WT-CFBE cells (Lu et al., 2019).

Despite the aforementioned reports, the study of interactions between cholesterol and CFTR protein has just begun. However, a few important factors must be considered. First, the ability to strictly control cholesterol levels in the plasma membrane serves as a key barrier in this research. Removal of too much cholesterol from the cell will lead to cell death, while removal of too little cholesterol from the cell will not dramatically affect the behavior of the protein of interest (i.e., CFTR). Second, although many studies used cholesterol-loaded MβCD for enrichment of cholesterol in the plasma membrane, this, too, is difficult to control accurately. Third, it is not clear how sensitive cholesterol in lipid rafts is to catalysis by CO or CE. Fourth, more mutant CFTR variants need to be tested and compared in order to identify the most significant modulation in CFTR channel function by cholesterol manipulation. Fifth, it will likely be necessary to combine multiple techniques, including molecular dynamic simulations, in order to identify the binding site(s) for cholesterol at CFTR and completely understand the direct interaction. The use of tools such as ALOD4 (the bacterial toxin anthrolysin O), which binds and traps accessible cholesterol in the outer leaflet of plasma membranes of intact cells without changing total cholesterol abundance (Endapally et al., 2019), would allow one to study the effects of trapped cholesterol on CFTR function without disrupting the cell plasma membrane. In addition, animal models that alter cholesterol metabolism could be useful tools for studying interactions between CFTR and cholesterol (Amsalem et al., 2018).

## Data Availability Statement

The raw data supporting the conclusions of this article will be made available by the authors, without undue reservation.

## Author Contributions

GC designed studies, generated and analyzed data for FRT cells, and wrote the manuscript. KC designed studies, generated and analyzed data for HBE cells, and reviewed the manuscript. KS contributed to cell culture for both FRT and HBE cells. SM and MK contributed to cell culture for HBE cells. NM was responsible for overall study design, review of all results, and editing the manuscript. All authors contributed to the article and approved the submitted version.

## Conflict of Interest

The authors declare that the research was conducted in the absence of any commercial or financial relationships that could be construed as a potential conflict of interest.
