# Supplementary material for: Alteration of Membrane Cholesterol Content Plays a Key Role in Regulation of Cystic Fibrosis Transmembrane Conductance Regulator Channel Activity
Source: Front Physiol. 2021 Jun 7;12:652513. doi: 10.3389/fphys.2021.652513 (PMC8215275; doi:10.3389/fphys.2021.652513)
Supplement: Supplementary file 1 [file Data_Sheet_1.docx]

**Cui et al., Frontiers Physiology MS ID #652513 Supplemental Figures and Legends, with Refs.**

**Supplementary Figure 1.** Representative short circuit current traces for WT- (A) and P67L-CFTR (B) activated with various concentrations of FSK after pre-treatment with 5 U/ml cholesterol oxidase at 37°C for 1 hour. FSK concentration are labeled as follows: a = 1 nM; b = 11 nM; c = 111 nM; d = 1 µM; e = 11 µM; f = 36 µM; and g = 71 µM. Currents were inhibited by 10 µM INH172 at the end of the experiment. Both FSK and INH172 were added in the apical side of the Ussing chamber.

**Supplementary Figure 2.** WT-CFTR expressed in *Xenopus* oocytes reacted to GlyH-101 differently without (control, A) *vs.* following MβCD treatment (B). Representative whole oocyte two-electrode voltage clamp (TEVC) current traces were recorded at V_m_ = −60 mV in ND96 solution (**A**, **B**) (1, 2). CFTR channels were activated by stimulation with 10 µM forskolin (FSK). After activation to steady state, oocytes were exposed to 5 μM GlyH-101 in the continuing presence of 10 µM FSK. (**A**) Control. (**B**) Oocytes were pretreated with 20 mM MβCD for 3 hours at room temperature. (**C**) Dose-response curves for GlyH-101-mediated inhibition of WT-CFTR were shifted to left in the MβCD pre-treatment group. Both traces were fit with the one site ligand binding equation in Sigmaplot 12.3. Concentrations for half maximal inhibition (IC_50_) are 1.5 µM for MβCD (pre-treatment group) and 4.2 µM for control (non-treatment group). n = 13 for control group; n = 9 for MβCD group. **, *P* < 0.01; ***, *P* < 0.001 compared to control.

1. Cui G, Hong J, Chung-Davidson YW, Infield D, Xu X, Li J, et al. An Ancient CFTR Ortholog Informs Molecular Evolution in ABC Transporters. *Dev Cell.* 2019;51(4):421-30 e3.

2. Cui G, and McCarty NA. Murine and human CFTR exhibit different sensitivities to CFTR potentiators. *American journal of physiology Lung cellular and molecular physiology.* 2015;309(7):L687-99.
